# Comparative effectiveness of aspirin for symptomatic venous thromboembolism prophylaxis in patients undergoing total joint arthroplasty, a cohort study

**DOI:** 10.1186/s12891-023-06750-x

**Published:** 2023-08-03

**Authors:** Verinder Sidhu, Helen Badge, Timothy Churches, Justine Maree Naylor, Sam Adie, Ian A Harris

**Affiliations:** 1 School of Clinical Medicine, UNSW Medicine and Health, UNSW Sydney, Sydney, NSW Australia; 2 Whitlam Orthopaedic Research Centre, Ingham Institute for Applied Medical Research, Liverpool, Australia; 3 Australian Catholic University, School of Public and Allied Health, North Sydney, 8–20 Napier Street 2069 Australia; 4 Institute of Musculoskeletal Health, School of Public Health, The University of Sydney, Sydney, NSW Australia

**Keywords:** Venous thromboembolism, Arthroplasty, Aspirin, Low-molecular Weight Heparin, Novel oral anticoagulants

## Abstract

**Background:**

This study compares the symptomatic 90-day venous thromboembolism (VTE) rates in patients receiving aspirin to patients receiving low-molecular weight heparin (LMWH) or direct oral anticoagulants (DOACs), after total hip (THA) and total knee arthroplasty (TKA).

**Methods:**

Data were collected from a multi-centre cohort study, including demographics, confounders and prophylaxis type (aspirin alone, LMWH alone, aspirin and LMWH, and DOACs). The primary outcome was symptomatic 90-day VTE. Secondary outcomes were major bleeding, joint related reoperation and mortality within 90 days. Data were analysed using logistic regression, the Student’s t and Fisher’s exact tests (unadjusted) and multivariable regression (adjusted).

**Results:**

There were 1867 eligible patients; 365 (20%) received aspirin alone, 762 (41%) LMWH alone, 482 (26%) LMWH and aspirin and 170 (9%) DOAC. The 90-day VTE rate was 2.7%; lowest in the aspirin group (1.6%), compared to 3.6% for LMWH, 2.3% for LMWH and aspirin and 2.4% for DOACs. After adjusted analysis, predictors of VTE were prophylaxis duration < 14 days (OR = 6.7, 95% CI 3.5–13.1, p < 0.001) and history of previous VTE (OR = 2.4, 95% CI 1.1–5.8, p = 0.05). There were no significant differences in the primary or secondary outcomes between prophylaxis groups.

**Conclusions:**

Aspirin may be suitable for VTE prophylaxis following THA and TKA. The comparatively low unadjusted 90-day VTE rate in the aspirin group may have been due to selective use in lower-risk patients.

**Trial Registration:**

This study was registered at ClinicalTrials.gov, trial number NCT01899443 (15/07/2013).

## Background

Venous thromboembolism (VTE) comprises deep venous thrombosis (DVT) and pulmonary embolism (PE) and is a serious complication following total hip arthroplasty (THA) and total knee arthroplasty (TKA). Multi-modal VTE prevention using early mobilization, regional anaesthesia, mechanical and chemoprophylaxis has helped to reduce the rates of VTE after THA and TKA [1, 2], however, there remains controversy as to the most effective and safest form of chemoprophylaxis [3–6].

Current guidelines recommend low-molecular weight heparin (LMWH), aspirin, direct oral anticoagulants (DOACs) or warfarin for chemoprophylaxis [3, 4, 7]. Each form of prophylaxis has advantages and disadvantages. Proponents of aspirin claim ease of administration, low surgical site bleeding risk and low cost, whereas proponents of LMWH, DOACs and warfarin claim greater effectiveness for VTE prevention compared to aspirin [3, 4]. Whilst there are clinical trials that have compared LMWH to DOACs [8], prospective trials comparing aspirin as a sole agent to LMWH and other forms of VTE prophylaxis (warfarin, DOACs) are lacking [5, 9, 10].

This study aims to compare the real-world safety and effectiveness of aspirin to LMWH and DOACs in reducing symptomatic 90-day VTE rates in patients undergoing elective THA or TKA for osteoarthritis. It was hypothesized that patients receiving aspirin would have equivalent rates of symptomatic VTE when compared to LMWH and DOACs.

## Methods

We used data collected from the Evidence Based Processes and Outcomes of Care (EPOC) Study. EPOC was a multi-center prospective observational cohort study aimed at investigating the association between rates of compliance with evidence-based guidelines for the prevention of VTE and infection and related outcomes for patients undergoing elective primary THA and TKA. Ethical approval was obtained from the nine relevant human research ethics committees and informed consent was obtained for each patient recruited.

The EPOC study collected data from patients aged 18 or older undergoing primary, elective THA or TKA for osteoarthritis at 19 participating high-volume arthroplasty centers throughout Australia between 2013 and 2015. High volume centers were defined as those performing greater than 275 THA and TKA procedures annually [11, 12]. Data collected by the EPOC study included baseline demographic and health data, acute-care data, patient reported outcomes and postoperative complications (including VTE and non-VTE complications) at 35, 90 and 365 days. Site coordinators screened and gained informed consent from participants prior to surgery; acute-care date were extracted from the medical records by site coordinators and researchers undertook a secondary audit of records at a later date. Participants were contacted by telephone by researchers at 90 days post-discharge to determine if postoperative complications (VTE, bleeding, reoperation) had occurred. If the patient reported a postoperative complication, this was verified through contact with the patient’s primary care physician or surgeon.

Baseline data in this study included age, sex, type of arthroplasty (THA or TKA), bilateral arthroplasty, body-mass index (BMI), American Society of Anesthesiologist’s (ASA) grade [13] and other factors known to affect the risk of VTE in patients undergoing arthroplasty, namely, current smoking status, prior history of VTE, concurrent history of cancer and the use of neuraxial (spinal or epidural) anesthesia [14].

Postoperative data included the type and duration of chemoprophylaxis used, the postoperative day that patients were first mobilized, the duration of mechanical prophylaxis used and whether patients underwent routine duplex ultrasound (DUS) for DVT. One participating institution and one surgeon at a second institution performed routine DUS for DVT postoperatively. Mechanical prophylaxis (calf compressors for the first 24 h, followed by graduated compression stockings) were universally used amongst all institutions. The decision on duration of prophylaxis and on whether to prescribe aspirin, LMWH, DOACs (rivaroxaban) or warfarin for VTE prophylaxis was based on surgeon preference and was not assigned by this study. Aspirin was administered at 100 mg, orally daily, dalteparin at 5000 IU subcutaneously, daily, enoxaparin at 40 mg, subcutaneously, daily and rivaroxaban at 10 mg, orally, once daily.

The primary outcome of this study was symptomatic VTE within 90 days of surgery (including infra-popliteal DVT). Patients at institutions where routine DUS was used were adjudicated to have a symptomatic DVT only if they also demonstrated persistent calf swelling, tenderness and pain. Not all DVTs diagnosed by routine DUS were considered to be symptomatic. No institution performed routine ventilation/perfusion scans or computer tomography pulmonary angiograms for the detection of PE. Secondary outcomes included non-VTE related complications within 90 days of surgery; major bleeding (bleeding resulting in readmission, re-operation or death), reoperation on the operated joint and death.

### Statistical analyses

Statistical analyses were carried out using the R Environment for Statistical Computing (version 3.6.1). A sample size calculation as not performed as this was a secondary analysis of data from the EPOC study, with the number of recruited patients already determined by the EPOC study and further recruitment not possible. Further, as a secondary aim was to provide an event rate for a subsequent cluster randomized controlled trial [15], the study also served as a pilot study.

Patients were classified into four groups based on the type of pharmacological prophylaxis used. These groups were patients who received (1) aspirin only, (2) LMWH (dalteparin or enoxaparin) only, (3) LMWH and aspirin either concurrently or sequentially and (4) DOACs. Baseline demographic, operative and postoperative data were tabulated and compared for each prophylaxis group. ASA classification was dichotomized into whether patients had an ASA > 2 or not, the time to mobilization postoperatively into whether this occurred by the end of day 1 and duration of chemoprophylaxis into whether patients received greater than or equal to 14 days or not. The cut-off of 14 days was chosen as this represents the current minimum recommended duration of prophylaxis from the most recent international guidelines [7]. Median duration of prophylaxis was also reported rather than mean as it was expected that this variable would not exhibit a normal distribution.

### Analyses for the primary outcome

The rate of symptomatic VTE for the entire cohort was described overall and categorized via type (pulmonary embolism or deep venous thrombosis) for each prophylaxis group. The median time to VTE diagnosis for each group was also described. Univariate analyses were used to identify predictors of VTE. Fisher’s exact test was used for categorical, dichotomous variables and logistic regression for categorical non-dichotomous variables and for continuous variables, with the outcomes reported using a point estimate (odds ratio – OR) and a 95% confidence interval.

Factors identified on univariate analysis with a p-value < 0.25 were entered into a backwards, stepwise multivariable logistic regression model (using the Akaike information criterion – AIC) to identify independent predictors of symptomatic VTE. As the exposure of interest, type of VTE prophylaxis was forced into the multivariable regression model. Known potential confounders of VTE (age, sex, BMI, smoking status, joint replaced and prior history of VTE) were forced into the model regardless of whether they produced a p-value of < 0.25 on univariate analyses.

Multiple imputation using chained equations was used to address missing values and model averaging from each individual imputed dataset, with pooled results was used for the multivariable analysis. As the objective of the study was to investigate symptomatic VTE only, routine DUS was omitted from the multivariable regression model.

### Sensitivity analyses for the primary outcome

A sensitivity analysis removing duration of VTE prophylaxis from the final model was performed to account for possible reverse causation whereby prophylactic agents are ceased at the time of VTE diagnosis (therefore shortening the duration of prophylaxis). The coefficients of the remaining variables were compared to those in the final model to determine if duration had affected the association between the remaining variables and VTE.

### Secondary outcomes

Secondary outcomes were compared by type of VTE prophylaxis used. Univariate analyses (logistic regression) were used to determine whether the type of VTE prophylaxis was associated with an increased risk of each secondary outcome. Multivariable regression was not performed for secondary outcomes.

## Results

There were 1867 out of 1904 patients enrolled in the prospective EPOC study who had complete follow-up data available at 90 days postoperatively (follow-up rate of 97%) and who provided informed consent. There were 40 patients with missing data for ASA (2%), 28 with missing data for whether routine DUS was used (2%), 15 with missing data for day first mobilized (1%), 10 with missing data for smoking status (0.6%), 10 with missing data for prophylaxis duration (0.5%), two with missing data for history of previous of VTE (0.1%) and one patient with missing data for type of anesthesia (0.05%).

Baseline demographic data for patients by VTE prophylaxis subgroup is listed in Table 1. There were an additional 77 patients (4%) who received warfarin, 4 (0.2%) who received unfractionated heparin and 7 (0.4%) who received no chemoprophylaxis. Due to the small numbers of patients in these groups, these patients were omitted from the analyses.

In the aspirin group, there were lower proportions of patients with a history of a prior VTE, undergoing TKA, undergoing bilateral arthroplasty, receiving neuraxial anesthesia and having an ASA grade more than 2, and a higher proportion of patients who were mobilized early and receiving greater than or equal to 14 days of VTE prophylaxis. There were no other significant differences in baseline demographic information.

The median duration of prophylaxis in the aspirin group was 42 days (interquartile range (IQR) 35 to 42 days), was 15 days (IQR 10 to 25 days) in the LMWH only group, was 23 days (IQR 11 to 42 days)in the LMWH and aspirin group and was 27 days (IQR 17 to 36) in the DOAC group.

**Table 1:** Demographic data by subgroup of venous thromboembolism prophylaxis used

	Aspirin Only (n = 365, 20%)	Low molecular weight heparin only (n = 762, 41%)	Low molecular weight heparin and aspirin (n = 482, 26%)	Direct oral anticoagulant (n = 170, 9%)	Total (n = 1867)
Age (yrs, mean, SD)	67 (*9.7*)	66 (*9.3*)	68 (*10.2*)	68 (*9.1*)	67.2 (9.7)
Gender (n, *%*)					
Male	168 (*46*)	322 *(42)*	240 (*50*)	79 (*47*)	1012 (*54*)
Female	197 (*54*)	440 (*58*)	242 (*50*)	91 (*53*)	855 (*46*)
Body-mass index (kg/m^2^, mean, SD)	31 (*6.4*)	31 (*6.9*)	31 (*6.0*)	30 (*5.7*)	31 (*6.4*)
Current Smoker (n, *%*)	29 (*8*)	76 (*10*)	36 (*8*)	10 (*6*)	155 (*8*)
ASA (n, %)					
< 3	255 (*72*)	532 (*71*)	304 (*64*)	120 (*71*)	1242 (*68*)
≥ 3	101 (*28*)	215 (*29*)	169 (*36*)	48 (*29*)	585 (*32*)
Type of Joint (n, *%*)					
Total hip arthroplasty	203 (*56*)	300 (*39*)	195 (*40*)	84 (*49*)	813 (*43*)
Total knee arthroplasty	162 (*44*)	462 (*61*)	287 (*60*)	86 (*51*)	1054 (*57*)
Bilateral Joint (n, *%*)	1 (0.*3*)	42 (*6*)	18 (*4*)	27 (*16*)	91 (*5*)
History of previous venous thromboembolism (n, %)	11 (*3*)	49 (*6*)	44 (*9*)	23 (*14*)	148 (*8*)
Spinal or Epidural Anaesthesia (n, %)	163 (*45*)	518 (*68*)	325 (*67*)	120 (*71*)	1178 (*63*)
Prophylaxis Duration ≥ 14 days (n, %)	330 (90)	496 (65)	341 (71)	145 (85)	1350 *(72)*
Prophylaxis Duration (median, IQR)	42 (*35–45)*	15 (*10–25*)	23 (*11–42*)	27 (*17–36*)	22 (*12–36*)
No. Patients Mobilised by end of Day 1 (n, %)	294 (*81*)	390 (*51*)	282 (*59*)	67 (*40*)	1077 (*58*)

### Primary outcome

The 90-day symptomatic VTE rate in the entire cohort was 2.7% (n = 50), with a pulmonary embolism rate of 1% (n = 19) and a deep venous thrombosis rate of 1.7% (n = 32). One patient had both a pulmonary embolus and a deep venous thrombosis. The VTE rate in the aspirin group was 1.6%, which was lower than the VTE rate in all other groups (3.6% for LMWH only, 2.3% for LMWH and aspirin and 2.4% for DOACs), however, this difference was not significant (Table 2). The median time to diagnosis for VTE was 7 days in the aspirin group, 7 days for the LMWH only group, 10 days for the LMWH and aspirin group and 8 days in the DOAC group.

**Table 2:** Primary and secondary outcomes

	Aspirin (n = 363)	Low molecular weight heparin (n = 758)	Low molecular weight heparin & aspirin (n = 478)	Direct oral anticoagulant (n = 170)	*p*
Any venous thromboembolism (n, *%*)	6 (*1.6*)	27 (*3.6*)	11 (*2.3*)	4 (*2.4*)	0.6
Pulmonary embolism (n, *%*)	1 (*0.3*)	13 (*1.7*)	4 (*0.8*)	0 (*0*)	0.3
Deep venous thrombosis (n, *%*)	5 (*1.4*)	15 (*2.0*)	7 (*1.5*)	4 (*2.4*)	1.0
Major bleeding within 90 days (n, %)	5 (*1.4*)	1 (*0.1*)	6 (*1.2*)	4 (*2.4*)	0.1
Joint-related reoperation within 90 days (n, %)	15 (*4.1*)	21 (*2.8*)	8 (*1.7*)	9 (*5.3*)	0.2
Death within 90 days (n, %)	2 (*0.5*)	3 (*0.4*)	0 (*0*)	0 (*0*)	0.7

Univariate analyses are displayed in Table 3. Factors associated with a significantly increased risk of VTE were a shorter duration of prophylaxis, TKA (compared to THA), mobilization occurring after postoperative day 1, a history of a previous VTE and increasing BMI.

**Table 3:** Univariate analyses for symptomatic venous thromboembolism within 90 Days

	Odds Ratio	95% Confidence Interval	*p*
Prophylaxis Type			
Aspirin	Reference		
Low molecular weight heparin only	2.2	0.96–5.9	0.08
Low molecular weight heparin and aspirin	1.4	0.5–4.1	0.5
Direct oral anticoagulant	1.4	0.4–5.1	0.6
Age (per year)	1.02	0.99–1.1	0.2
Body-mass index (per kg/m^2^)	1.05	1.01–1.1	0.01
Female Sex	1.4	0.8–2.6	0.3
ASA classification > 2	1.4	0.8–2.6	0.2
Duration < 14 Days	6.6	3.5–13.1	< 0.001
Total knee arthroplasty	3.2	1.6–7.1	< 0.001
Mobilised by Day 1	0.5	0.3–0.9	0.01
History of previous venous thromboembolism	2.3	0.9–5.0	0.05
Bilateral arthroplasty	1.7	0.4–4.9	0.3
Neuraxial anaesthesia used	1.7	0.9–3.5	0.1
Current smoker	0.5	0.1–1.7	0.4

ASA = American Society of Anesthesiologists

Factors with a p-value < 0.25 on univariate analysis that were entered into the multivariable logistic regression model were age, BMI, joint replaced (hip or knee), history of a previous VTE, ASA, neuraxial anesthesia, mobilization by day 1 and whether patients received more than or equal to 14 days of prophylaxis. Only prophylaxis duration less than 14 days, BMI, TKA, mobilization after day 1 and previous VTE remained in the final model as predictors of VTE. Receiving less than 14 days of prophylaxis (OR = 6.7, 95% CI 3.5 to 13.1, p < 0.001) and previous VTE (OR = 2.4, 95% CI 1.01 to 5.8, p = 0.05) remained as the only statistically significant predictors of 90-day symptomatic VTE in the final model (Table 4).

**Table 4:** Final adjusted multivariable model for symptomatic venous thromboembolism within 90 Days ^†^

	Odds Ratio	95% Confidence Interval	*p*
Prophylaxis Type			
Aspirin	Reference		
Low molecular weight heparin only	0.8	0.3–2.2	0.7
Low molecular weight heparin and aspirin	0.6	0.2–1.7	0.3
Direct oral anticoagulant	0.8	0.2–3.1	0.7
Duration < 14 days	6.7	3.5–13.0	< 0.001
History of previous venous thromboembolism	2.4	1.01–5.8	0.05
Body-mass index (per kg/m^2^)	1.03	0.99–1.1	0.1
Total knee arthroplasty	2.1	0.9–4.5	0.06
Mobilised by Day 1	0.6	0.3–1.1	0.1

^†^ Akaike information criterion (AIC) used to determine final model of best fit

### Sensitivity analyses

Removing duration or routine DUS from the final model did not alter the coefficients of the remaining variables by more than 10%.

### Secondary outcomes

Secondary outcomes are shown in Table 2. The incidence of major bleeding at 90 days was 0.9% (n = 17) and the joint-related reoperation rate at 90 days was 3%. There were five patients who died within 90 days of surgery (0.3%). Four patients died from respiratory failure (non-pulmonary embolism related) and one from a traumatic brain injury. There were no significant differences found in the rates of secondary outcomes by type of VTE prophylaxis received.

## Discussion

Despite the low symptomatic VTE rate seen in the aspirin group on unadjusted analysis (1.6%), there was no significant association between prophylaxis type and VTE after adjusting for confounders. The multivariable analysis suggested that the low VTE rate in the aspirin group was due to a lower baseline VTE risk in these patients (lower BMI, lower previous VTE rate and shorter time to mobilization).

The strengths of this study include data collection across multiple high-volume institutions, increasing the generalizability of the results and reflecting real-world practice, and the inclusion of parameters that have been omitted from previous studies such as duration of prophylaxis and time to mobilization. Further, all primary and secondary outcomes were verified through contact with treating clinicians and hospitals.

This study also has several limitations. First, the study was likely underpowered to detect a difference for the primary outcome, given the low incidence of VTE within each prophylaxis group. Reliable conclusions cannot be drawn regarding the effectiveness of aspirin from this study due to the low included sample size compared to existing published randomized and observational studies. Second, there was considerable heterogeneity of prophylaxis protocols among participating institutions which limited the ability to perform a direct comparison between each form of prophylaxis. Third, its retrospective, observational nature increased the risk of selection bias. It is likely that aspirin was prescribed to patients with a lower baseline VTE risk in this study and that LMWH and DOACs were prescribed to higher risk patients.

Selection bias may also be a contributing factor to the low rates of VTE reported with aspirin use compared to other forms of prophylaxis in other observational and registry-based studies [16–27]. Eight recent observational studies [16–18, 23–27] and five retrospective registry based or health insurance database studies [19–22, 28] have reported either decreased or comparable symptomatic VTE rates for aspirin compared to LMWH, warfarin or DOACs, for both low and high-risk patients [23, 25]. However, like the current study, the allocation of patients to VTE prophylaxis groups was at the discretion of treating surgeons and the number of patients receiving aspirin was considerably lower than the number of patients receiving other forms of anticoagulation for each of these studies [16, 18–26, 28].

Although several of the previous observational studies attempted to adjust for potential confounders with multivariable analyses, these have produced inconsistent results [19–25]. Four studies demonstrated that aspirin did not remain as an independent protective factor against VTE after adjusted analyses [19–21, 24], one study demonstrated it was non-inferior to other forms of VTE prophylaxis [22] and two studies reported that aspirin remained as an independent protective factor against symptomatic VTE [23, 25]. The authors of each study acknowledged that selection bias and a failure to include all known confounders were limitations in their methodology, with no study including time to mobilization or duration of VTE prophylaxis in their adjusted analyses [29, 30].

The effect of early mobilization as a confounder has not been included in the majority of observational studies investigating the efficacy of aspirin for VTE prophylaxis following THA and TKA. Early mobilization has long been associated with lower VTE rates [7, 31] and given the advent of day-only arthroplasty and accelerated recovery protocols, this is an important confounding factor that needs consideration in studies investigating the efficacy of aspirin for VTE prophylaxis after THA and TKA.

The results from this study and previous observational studies highlight the limitations associated with relying on retrospective evidence regarding the efficacy and safety of aspirin when used for VTE prophylaxis for all patients following THA or TKA. It is likely that patients with a low baseline risk of VTE were more likely to be allocated to aspirin groups (as occurred in our study), leading to lower symptomatic VTE rates compared to other forms of prophylaxis (selection bias). This may have been the result of risk stratification protocols implemented by individual surgeons at participating hospitals, which have been proposed as an alternate strategy for VTE prophylaxis rather than prescribing one agent for all patients [32].

Balancing all confounding factors is more reliable through randomization. Only three randomized trials have investigated the use of aspirin for symptomatic VTE prophylaxis following THA and TKA within the last 20 years. The largest study (n = 9711), published in 2022, demonstrated that enoxaparin was superior to aspirin in preventing all symptomatic VTE (including below knee DVT, above knee DVT and PE) within 90 days following THA or TKA performed for a diagnosis of osteoarthritis [33]. In the remaining two studies, extended aspirin use was found to be effective compared to rivaroxaban or LMWH. However, patients initially received either LMWH (for 10 days) or rivaroxaban (for 5 days) prior to being randomized for extended prophylaxis, which does not reflect the current use of aspirin in many institutions[22–25], where it is most commonly used as a sole agent [9, 10].

Evidence from observational studies has led to an increase in the use of aspirin use over the last decade [26, 34, 35] and it is now used as the primary prophylactic agent in greater than 95% of patients receiving THA or TKA throughout the United States according to a survey of members of the American Association of Hip and Knee Surgeons [35]. Due to the inherent inability of observational studies to effectively balance all confounders (known and unknown), guidelines should place more emphasis on randomized studies when recommending chemoprophylaxis agents.

## Conclusions

Although interpretation of this study’s results is limited by its small sample size, aspirin use was associated with a low risk of symptomatic VTE compared to LMWH and DOACs. On adjusted analyses, aspirin was not associated with a decreased risk of VTE, which was likely due to its use in patients with a low baseline risk of VTE, a finding common to other observational studies. Evidence from prospective randomized trials should be prioritized in guiding recommendations regarding the safety and efficacy of aspirin immediately following THA and TKA, for both low and high-risk patients.

## Data Availability

The datasets used and/or analysed during the current study are available from the corresponding author on reasonable request.
